# Quantifying non-ergodicity of anomalous diffusion with higher order moments

**DOI:** 10.1038/s41598-017-03712-x

**Published:** 2017-06-20

**Authors:** Maria Schwarzl, Aljaž Godec, Ralf Metzler

**Affiliations:** 1 Institute for Physics & Astronomy, University of Potsdam, 14476 Potsdam-Golm, Germany; 2 National Institute of Chemistry, 1000 Ljubljana, Slovenia

## Abstract

Anomalous diffusion is being discovered in a fast growing number of systems. The exact nature of this anomalous diffusion provides important information on the physical laws governing the studied system. One of the central properties analysed for finite particle motion time series is the intrinsic variability of the apparent diffusivity, typically quantified by the ergodicity breaking parameter EB. Here we demonstrate that frequently EB is insufficient to provide a meaningful measure for the observed variability of the data. Instead, important additional information is provided by the higher order moments entering by the skewness and kurtosis. We analyse these quantities for three popular anomalous diffusion models. In particular, we find that even for the Gaussian fractional Brownian motion a significant skewness in the results of physical measurements occurs and needs to be taken into account. Interestingly, the kurtosis and skewness may also provide sensitive estimates of the anomalous diffusion exponent underlying the data. We also derive a new result for the EB parameter of fractional Brownian motion valid for the whole range of the anomalous diffusion parameter. Our results are important for the analysis of anomalous diffusion but also provide new insights into the theory of anomalous stochastic processes.

## Introduction

Superresolution microscopy allows unprecedented insight into the motion of fluorescently labelled, single molecules in complex liquid environments and even inside living biological cells. The observed tracer dynamics also reveals new insights into the physical properties of the systems and thus provides a handle for the modelling of followup processes such as molecular reactions in the system. Concurrently, due to ever increasing computational power, large scale simulations uncover longer and longer time windows of the atomistic or coarse grained dynamics in molecular systems^1–6^.

In complex systems such as living biological cells one often observes systematic deviations of the tracer dynamics from Brownian motion. Thus, anomalous diffusion characterised by the power-law scaling

1 $$\langle {x}^{2}(t)\rangle \simeq {K}_{\alpha }{t}^{\alpha }$$

of the mean squared displacement (MSD) emerges, where *K*_*α*_ is the generalised diffusion coefficient of dimension cm^2^/sec^*α*^. According to the magnitude of the anomalous diffusion exponent *α* one distinguishes subdiffusion for 0 < *α* < 1 and superdiffusion for *α* > 1^7, 8^. Subdiffusion, for instance, was observed for the motion of tracer particles inside living biological cells^9–15^, in artificially crowded^16–18^ and structured^19–22^ liquids, in pure and protein-crowded lipid bilayer systems^23–29^, as well as in groundwater systems^30^. Superdiffusion occurs in the presence of active motion, for instance, in living biological cells^31–34^ or due to bulk-surface exchange^35, 36^.

The MSD (1) is obtained as the average of the squared particle position over an ensemble of particles at a fixed time *t*. In many single particle tracking studies and large scale computer simulations few but long time series *x*(*t*) of the tracer particle position are available. These are typically evaluated in terms of the time averaged MSD

2 $$\overline{{\delta }^{2}({\rm{\Delta }})}=\frac{1}{t-{\rm{\Delta }}}{\int }_{0}^{t-{\rm{\Delta }}}{[x(t^{\prime} +{\rm{\Delta }})-x(t^{\prime} )]}^{2}dt^{\prime} ,$$

where Δ is called the lag time and *t* is the overall length of the time series^4, 37^. In the Boltzmann-Khinchin sense we call a stochastic system ergodic when the time averaged MSD (2) converges to the MSD (1) in the long measurement time limit: ${\mathrm{lim}}_{t\to \infty }\overline{{\delta }^{2}({\rm{\Delta }})}=\langle {x}^{2}({\rm{\Delta }})\rangle$. For certain anomalous diffusion processes this equality no longer holds, and we observe a so-called weak ergodicity breaking: ${\mathrm{lim}}_{t\to \infty }\overline{{\delta }^{2}(\Delta )}\ne \langle {x}^{2}({\rm{\Delta }})\rangle$ ^4, 37–44^.

For finite measurement times even for ergodic processes the time averaged MSD (2) will exhibit more or less pronounced amplitude variations around the mean

3 $$\langle\overline{{\delta }^{2}({\rm{\Delta }})}\rangle =\frac{1}{N}\sum _{i=1}^{N}\overline{{\delta }_{i}^{2}({\rm{\Delta }})}$$

taken over *N* garnered trajectories^4, 37, 38^. Defining the dimensionless variable $\xi =\overline{{\delta }^{2}({\rm{\Delta }})}/\langle \overline{{\delta }^{2}({\rm{\Delta }})}\rangle$ the variations of $\overline{{\delta }^{2}({\rm{\Delta }})}$ around $\langle \overline{{\delta }^{2}({\rm{\Delta }})}\rangle$ are then typically characterised in terms of the variance of *ξ*,

4 $${\rm{EB}}({\rm{\Delta }})=\langle {\xi }^{2}\rangle -\mathrm{1,}$$

the ergodicity breaking parameter^4, 37, 38, 45, 46^. The EB parameter has become a widely used tool to quantify the trajectory-to-trajectory fluctuations in single particle tracking. Note that in some papers EB is defined only in the limit Δ/*t* → 0, however, we here use it as a measure for the spread *ξ* also at finite Δ/*t* ratios. The canonical Brownian motion is characterised by the scaling EB = 4/3 × (Δ/*t*) of the EB parameter in the limit Δ/*t* → 0^4, 45^, see ref. 47 for the full expression.

Here we study in detail the full distribution *ϕ*(*ξ*) of the amplitude fluctuations of the time averaged MSD. For the most popular anomalous diffusion processes we demonstrate that while the EB parameter is an important measure for the amplitude of these fluctuations, in many cases it is not sufficient to adequately characterise the distribution *ϕ*(*ξ*). Namely, in many cases *ϕ*(*ξ*) is significantly skewed, that is, asymmetric around its mean. In this case higher order moments should be used to complement the EB parameter. Specifically, we here analyse the skewness

5 $$\gamma =\langle{(\frac{\xi -1}{\sqrt{\langle {\xi }^{2}\rangle -1}})}^{3}\rangle =\frac{\langle {\xi }^{3}\rangle -3\langle {\xi }^{2}\rangle +2}{{(\langle {\xi }^{2}\rangle -1)}^{3/2}},$$

where the denominator represents the standard deviation *σ* = (〈*ξ* ^2^〉 − 〈*ξ*〉^2^)^1/2^ = EB^1/2^ of the amplitude scatter of *ξ* and we used the fact that 〈*ξ*〉 = 1 by definition. When *γ* = 0 the distribution is symmetric, for negative/positive *γ* it is skewed to the left/right. When |*γ*| is larger than unity the distribution is considered significantly skewed. As we will see, this is frequently the case for the commonly used anomalous diffusion models, in particular for fractional Brownian motion (FBM), for which a symmetric Gaussian distribution for *ϕ*(*ξ*) was previously suggested. We also investigate in detail the kurtosis

6 $${\mathcal{K}}=\langle {(\frac{\xi -1}{\sqrt{\langle {\xi }^{2}\rangle -1}})}^{4}\rangle =\frac{\langle {\xi }^{4}\rangle -4\langle {\xi }^{3}\rangle +6\langle {\xi }^{2}\rangle -3}{{\langle {\xi }^{2}\rangle }^{2}-2\langle {\xi }^{2}\rangle +1},$$

which is a measure for the outliers of the scatter distribution *ϕ*(*ξ*). Alternatively in literature also the non-Gaussianity measure is used which involves the fourth time averaged moment^4^. As a reference process the analysis of the TAMSD of normal Brownian motion is discussed in ref. 47 in detail. It can be recovered as the special case *α* = 1 of our anomalous diffusion models. We note that *ϕ*(*ξ*) provides meaningful information even for relatively sparse data, and it can be reliably used to infer the physical character of the stochastic process underlying the observed data^48, 49^.

In the following section II we study the amplitude scatter for FBM at finite values of Δ/*t* and show, in particular, that the EB parameter varies smoothly as function of the anomalous diffusion exponent *α*. We also investigate the skewness and kurtosis for FBM and demonstrate the relevance to consider higher order moments. Section III is devoted to the subdiffusive continuous time random walk (CTRW), for which *ϕ*(*ξ*) is naturally skewed, as it has a finite value at *ξ* = 0. In addition to the standard CTRW we also quantify the shape parameters of *ϕ*(*ξ*) of ageing CTRW processes. Heterogeneous diffusion processes (HDP) with their systematic, quenched variation of the diffusion coefficient are then studied with respect to the skewness and kurtosis in section IV. Finally, in section V we draw our conclusions and argue, why this analysis of additional data inference techniques is important for a reliable quantitative and physical analysis of stochastic data. In the appendix some mathematical details are presented.

## Results

### Fractional Brownian Motion

FBM is one of the most widely used anomalous diffusion processes. For instance, it is a standard model for stock market dynamics^50^, and it is used as a polymer model^51^. It also describes the effective dynamics of single file diffusion^52^. Moreover, it is commonly used to model single particle diffusion experiments^12, 16, 17, 53–56^ in living cells. Its physical relevance stems from the fact that it corresponds to the overdamped limit of the fractional Langevin equation associated with particle motion in viscoelastic environments^4, 57, 58^.

FBM was formulated in 1968 by Mandelbrot and van Ness^59^ as a family of Gaussian random functions. We note that a similar process was introduced by Kolmogorov in 1940^60^. FBM is conveniently defined in terms of the Langevin equation

7 $$\frac{{\rm{d}}x(t)}{{\rm{d}}t}={\zeta }_{\alpha }(t),$$

where *ζ*_*α*_(*t*) represents fractional Gaussian noise, defined in terms of the Gaussian yet power-law correlated random noise *ζ*_*α*_(*t*) of zero mean and two-time correlation

8 $$\langle {\zeta }_{\alpha }({t}_{1}){\zeta }_{\alpha }({t}_{2})\rangle \sim 2{K}_{\alpha }\frac{\alpha }{2}(\alpha -1){|{t}_{1}-{t}_{2}|}^{\alpha -2},$$

for *t*_1_, *t*_2_ > 0 and *t*_1_ ≠ *t*_2_^45, 61–63^ The anomalous diffusion exponent *α* used here relates to the Hurst exponent, often encountered in the discussion of FBM, via *α* = 2*H*. Due to the sign of the factor (*α* − 1) subdiffusive FBM for 0 < *α* < 1 is anti-correlated (antipersistent), i.e., more erratic and with a smaller span than Brownian motion. Conversely, positively correlated (persistent) motion occurs in the range 1 < *α* < 2. In the limit *α* = 2 the steps are completely correlated and ballistic motion is recovered.

A sample path of FBM is generated analogously to Brownian motion in the form

9 $$x(t)={\int }_{0}^{t}{\zeta }_{\alpha }(t^{\prime} )dt^{\prime} \mathrm{.}$$

There are several ways to simulate fractional Gaussian noise, including the Hosking method^64^, which we used to generate our simulation paths. Accordingly the path is generated as the sum of a fractional Gaussian noise realisation in that every step is explicitly calculated recursively using the entire path history. The method is defined in detail in ref. 65.

### Ergodicity breaking parameter

FBM as well as ordinary Brownian motion is known to be ergodic in the Boltzmann-Khinchin sense in that ${\mathrm{lim}}_{{\rm{\Delta }}/t\to 0}\overline{{\delta }^{2}(\Delta )}=\langle {x}^{2}({\rm{\Delta }})\rangle$ ^45, 61^. Note, however, that for FBM and the related fractional Langevin equation motion transiently non-ergodicity and ageing effects may occur^18, 61, 66, 67^. Moreover the mean time averaged MSD $\langle \overline{{\delta }^{2}}\rangle$ equals the ensemble averaged MSD at all lag times Δ = *t* ^45^. The EB parameter of FBM was proposed to split up in the tripartite scaling law^45^

10 $${\rm{E}}{\rm{B}}=\{\begin{array}{cc}{\int }_{0}^{{\rm{\infty }}}{[{(1+\tau )}^{\alpha }+|1-\tau {|}^{\alpha }-2{\tau }^{\alpha }]}^{2}{\rm{d}}\tau \times \frac{{\rm{\Delta }}}{t}, & 0 < \alpha  < 3/2\\ 2{\alpha }^{2}{(\alpha -1)}^{2}\times \frac{{\rm{\Delta }}}{t}\,{\rm{l}}{\rm{n}}\,t, & \alpha =3/2\\ (\frac{4}{2\alpha -3}-\frac{4}{2\alpha -2}){(\frac{\alpha }{2})}^{2}{(\alpha -1)}^{2}\times {(\frac{{\rm{\Delta }}}{t})}^{4-2\alpha }, & 3/2 < \alpha  < 2\end{array}.$$

In this expression we see that the EB parameter converges to zero as $\simeq {\rm{\Delta }}/t$ proportionally to the Brownian case as long as *α* < 3/2. For *α* > 3/2 a slower decay $\simeq {({\rm{\Delta }}/t)}^{4-2\alpha }$ is observed.

**Figure 1:**
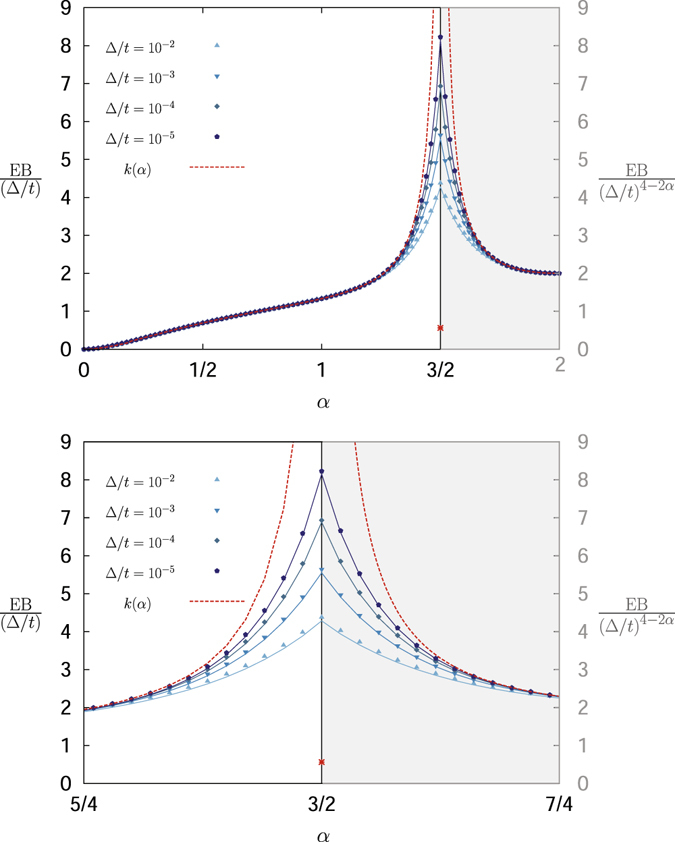
EB parameter of FBM divided by the respective scaling laws with respect to Δ/*t* for the regimes 0 < *α* < 3/2 and 3/2 < *α* < 2 according to Eq. (10). The behaviour according to Eq. (10) from^45^ is represented by the dashed red lines and the red symbol at *α* = 3/2: it shows a distinct divergence at *α* = 3/2. Our analytical result (12) based on a less severe approximation is represented by the symbols and the full line corresponding to the numerical evaluation of the integral (11), calculated via the trapezoidal rule with integration step 10^−3^. No divergence remains, and in fact a continuous albeit non-smooth behaviour is revealed. The agreement between our approximation (12) and the numerical evaluation of the full expression (11) remains quite good even for relatively large values of Δ/*t*. Note the left and right ordinates referring to the cases 0 < *α* < 3/2 and 3/2 < *α* < 2.

We also see that the result (10) includes a divergence at the critical point *α* = 3/2^45^, as shown in Fig. 1. Here we present the exact analytical result valid for any Δ/*t* based on a systematic, strictly converging series expansion (see Methods). In particular, we prove that in the limit ${\rm{\Delta }}/t\ll 1$ EB does not display any discontinuity at *α* = 3/2.

To show this let us reconsider the full expression of the EB parameter without approximation^45^,

11 $${\rm{EB}}=\frac{{{\rm{\Delta }}}^{-2\alpha }}{{(t-{\rm{\Delta }})}^{2}}{\int }_{0}^{t-{\rm{\Delta }}}(t-{\rm{\Delta }}-t^{\prime} ){[{(t^{\prime} +{\rm{\Delta }})}^{\alpha }+|t^{\prime} -{\rm{\Delta }}{|}^{\alpha }-2{t^{\prime} }^{\alpha }]}^{2}{\rm{d}}t^{\prime} \mathrm{.}$$

As detailed in Methods, this expression can be modified such that the consistent approximation ${\rm{\Delta }}/t\ll 1$ to leading order yields the FBM EB parameter

12 $${\rm{E}}{\rm{B}}\simeq \frac{{\rm{\Delta }}}{t}({k}_{1}(\alpha )\frac{{({\rm{\Delta }}/t)}^{3-2\alpha }-(2\alpha -2)}{2\alpha -3}+{k}_{2}(\alpha )),$$

where the prefactors are defined by

13 $${k}_{1}(\alpha )=\frac{{\alpha }^{2}}{2}(\alpha -\mathrm{1)}$$

and

14 $${k}_{2}(\alpha )=\frac{{\alpha }^{2}}{5}{(\alpha -1)}^{2}+\frac{8\alpha +8}{2\alpha +1}-\frac{8}{\alpha +1}+4\alpha (\alpha -1)(\frac{1}{3}-\frac{1}{\alpha +3}).$$

This result is clearly different from Eq. (10) and shows that the leading scaling behaviour suggested in ref. 45 is only assumed sufficiently far away from the point *α* = 3/2, with a quantitative dependence on Δ/*t*. As *α* approaches *α* = 3/2 the EB parameter according to expressions (12), (13), and (14) converges to the same value, and at *α* = 3/2 a linear term with a logarithmic correction in Δ/*t* emerges due to the exact limit

15 $$\mathop{{\rm{l}}{\rm{i}}{\rm{m}}}\limits_{\alpha \to 3/2}\frac{{({\rm{\Delta }}/t)}^{3-2\alpha }-1}{2\alpha -3}=-\,{\rm{l}}{\rm{n}}(\frac{{\rm{\Delta }}}{t}).$$

Hence, at *α* = 3/2 the EB parameter assumes the form

16 $${\rm{E}}{\rm{B}}=\frac{{\rm{\Delta }}}{t}(-\frac{9}{16}\,{\rm{l}}{\rm{n}}\,\frac{{\rm{\Delta }}}{t}+\frac{539}{240}).$$

The behaviour according to expressions (12), (13), and (14) is demonstrated in Fig. 1 in comparison with the numerically calculated full expression (11) of the EB parameter. Moreover, Fig. 1 confirms that the result of ref. 45 is only valid for *α* values, that are sufficiently far away from the point *α* = 3/2. Different values of the ratio Δ/*t* are shown and exhibit excellent agreement with our results. Only when we consider small values of Δ/*t* the scaling around the point *α* = 3/2 is somewhat off the leading order expansion obtained above. More specifically, in the range 0 < *α* < 3/2 the scaling of EB is $\simeq {\rm{\Delta }}/t$, hence the respective prefactor can be calculated by EB/(Δ/*t*). Analogously for 3/2 < *α* < 2 the prefactor is EB/(Δ/*t*)^4−2*α*^ and at exactly *α* = 3/2 it is EB/(Δ/*t* × ln*t*). We conclude that our analytical approximation (12) represents the behaviour of the EB parameter of FBM to numerically sufficient accuracy.

**Figure 2:**
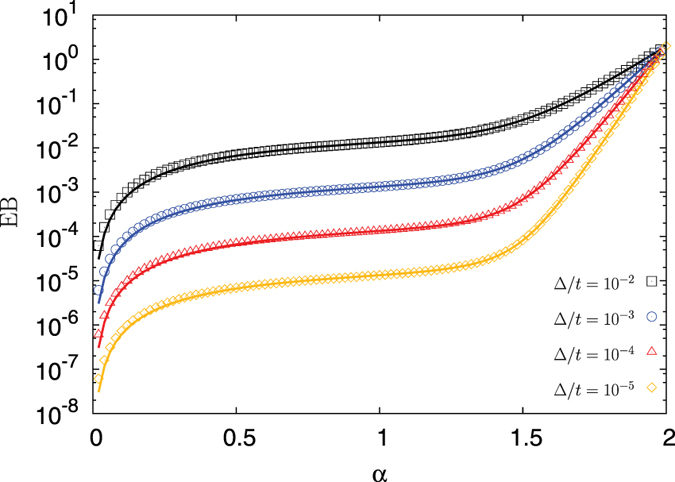
Analytical result (12) for the EB parameter of FBM, represented by the solid lines. The results of the numerical integrations of (11) are represented by the symbols and are calculated via the trapezoidal rule with integration step 10^−3^. Very good agreement is observed over the entire range of *α*.

Figure 2 shows the EB parameter as a function of the anomalous diffusion exponent for different ratios Δ/*t* of lag time to measurement time. We observe an excellent agreement between our analytical approximation (12) and the numerical result for the full integral (11). In particular, the value of EB is continuous across the value *α* = 3/2. Further one can see the transition in the scaling. Up to *α* = 3/2 the curves are equidistant due to the proportionality of EB to Δ/*t*. For larger values of *α* the curves progressively approach each other until the ballistic limit *α* = 2 where they reach the value EB = 2.

The continuity of EB as function of *α* and the approximative result for EB and its good agreement with the exact result are our first main finding.

### Skewness and kurtosis of the amplitude scatter distribution

**Figure 3:**
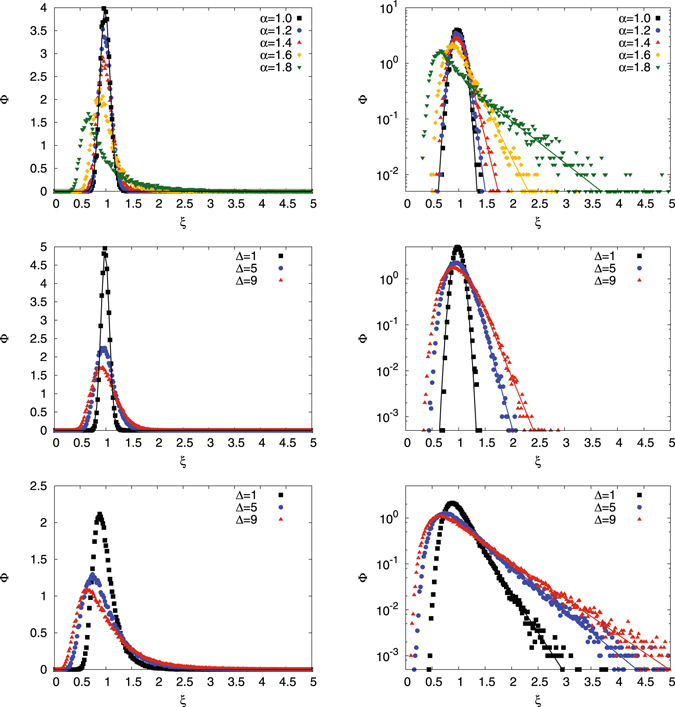
Amplitude scatter distributions *ϕ*(*ξ*) for FBM. Top: different values of the anomalous diffusion exponent, with *t* = 2^6^ and Δ = 1. The case of Brownian motion with *α* = 1 is shown with a Gaussian fit. Middle: different lag times for *α* = 0.6 and *t* = 2^6^. A Gaussian fit for the short lag time Δ = 1 shows nice agreement with the data, whereas at longer Δ the asymmetry of *ϕ*(*ξ*) becomes obvious in the semi-log scale on the right. Bottom: different lag times for *α* = 1.6 and *t* = 2^6^. High asymmetry of *ϕ*(*ξ*) is observed. In the semi-log scale on the right the tails are fitted exponentially.

As stated before, for finite measurement times the time averaged MSD of a single trajectory of any stochastic process is a random variable, whose distribution *ϕ*(*ξ*) provides information about the underlying stochastic process^4, 49, 68^. The result for *ϕ*(*ξ*) for FBM is depicted in Fig. 3. The top panels show the scatter distribution for different values of the anomalous diffusion exponent *α*, in the middle and at the bottom rows the scatter distribution is shown for different lag times Δ for the case of subdiffusion and superdiffusion with *α* = 0.6 and *α* = 1.6 respectively. The left panels show the data in linear scales whereas the right panels use a semi-logarithmic scale. The tails of the distributions are fitted by exponentials. The special case of Brownian motion with *α* = 1 is fitted by a Gaussian distribution in the top panel.

For FBM the amplitude scatter distribution *ϕ*(*ξ*) was shown to be approximately Gaussian at sufficiently short lag times for subdiffusive FBM–the underlying argument was based on the assumption that for small Δ any two displacements do not overlap for successive time intervals^49^. It was also shown that *ϕ*(*ξ*) becomes highly asymmetric for larger Δ^49^. As shown in Fig. 3 the Gaussian approximation indeed holds in the subdiffusive regime with 0 < *α* < 1 for Δ = 1. However, already for Δ = 5 the distribution *ϕ*(*ξ*) shows a pronounced asymmetry. In the superdiffusive case shown in the bottom panel of Fig. 3 this asymmetry is much more pronounced, and even in the case Δ = 1 the shape is obviously far beyond any Gaussian approximation. In all analysed cases the exponential tail

17 $$\varphi (\xi )\simeq \exp (-c\xi )$$

appears to capture the behaviour well.

**Figure 4:**
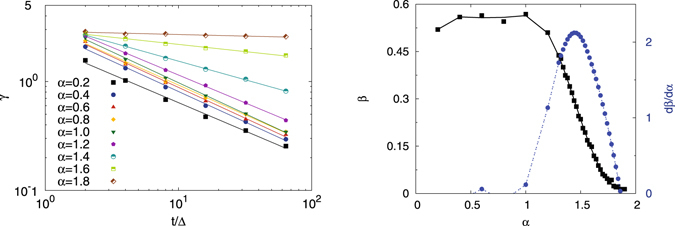
Skewness parameter of FBM as a function of the ratio *t*/Δ of the measurement and lag times from simulations evaluated at fixed Δ = 1, averaged over 10^5^ trajectories. Right: scaling exponent *β* of the skewness (squares) and the respective derivative d*β*/d*α* (circles). Note the left and right ordinates in the right panel.

**Figure 5:**
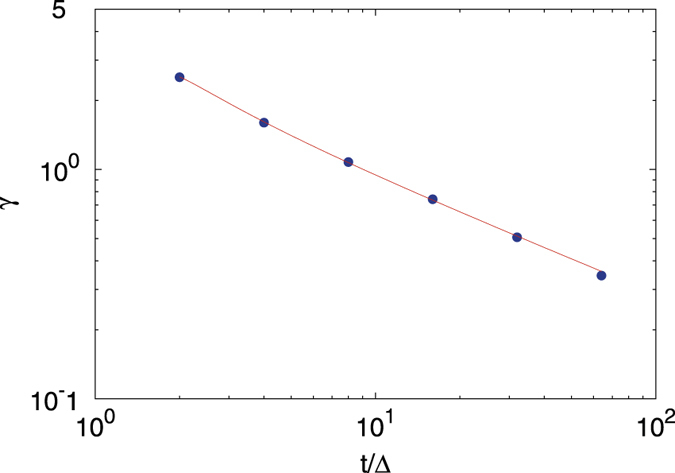
Numerical evaluation of the skewness of FBM for the special case *α* = 1 (blue dots). The solid line represents the analytic result from^47^.

We quantify the asymmetry of *ϕ*(*ξ*) by using the skewness parameter (5), which by definition is a function of the ratio of the lag time versus the measurement time as well as the anomalous diffusion exponent *α*. A symmetric distribution is characterised by a zero-valued skewness. We consider a distribution significantly skewed when the absolute value of the skewness parameter exceeds unity. Depending on the sign of the skewness a distribution can be skewed to the left or right. The skewness parameter evaluated from our simulations is shown in Fig. 4. For the special case of Brownian motion the explicit expression is known from^47^. The comparison to simulated data can be seen in Fig. 5, showing excellent agreement. Although the explicit formula for the skewness, apart from this special case, is unknown, the data can be tentatively fitted to a power-law in the ratio *t*/Δ of the measurement and lag times (the data are obtained for the fixed lag time Δ = 1) in the form

18 $$\gamma (t/{\rm{\Delta }})\simeq {(\frac{{\rm{\Delta }}}{t})}^{\beta }.$$

The scaling exponent *β* and its derivative *dβ*/*dα* are shown in the right panel of Fig. 4. For subdiffusive FBM the value of *β* remains approximately constant at around 1/2 for the entire subdiffusive range 0 < *α* < 1, leading to a rapid decay of the skewness as function of *t*/Δ, and thus an approximate validity of the Gaussian distribution for *ϕ*(*ξ*) proposed in ref. 49. In particular, in this analysis the Brownian limit *α* = 1 does not appear distinguished. However, once we reach the superdiffusive domain 1 < *α* < 2 the exponent *β* exhibits a distinct decay to zero. This means that for more superdiffusive FBM the asymmetry of *ϕ*(*ξ*) is a long-ranging characteristic of the process. We also note that the variation of *β* with the anomalous diffusion exponent becomes most delicate at the value *α* = 3/2, at which the (Δ/*t*)-scaling of the EB parameter crosses over, see Eq. (12).

**Figure 6:**
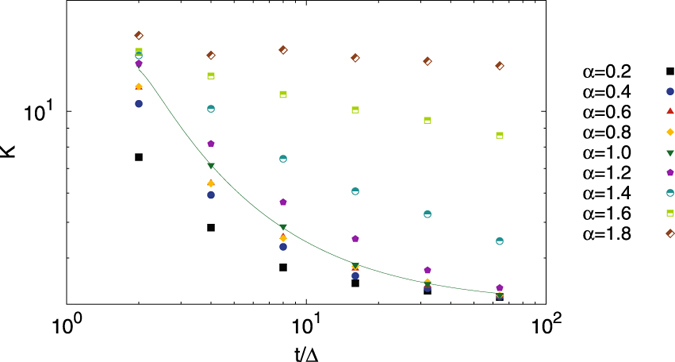
Kurtosis of FBM as function of the ratio *t*/Δ at fixed Δ = 1, averaged over 10^5^ trajectories. The solid line represents the analytical result for Brownian motion (*α* = 1) from^47^. The value *α* = 3 corresponds to a Gaussian distribution.

Figure 6 further analyses these observations in terms of the kurtosis. For the subdiffusive domain 0 < *α* < 1 the kurtosis converges relatively quickly to the expected value ${\mathcal{K}}=3$ for a Gaussian amplitude scatter distribution. Even for *α* = 1.2 the deviation is moderate. In contrast to this, for larger values of *α* the kurtosis converges significantly more slowly and assumes larger values, thus pointing at increasingly large outliers in the superdiffusive domain.

The strong asymmetry of the amplitude scatter distribution for FBM quantified in terms of the skewness and the kurtosis is our second main result.

### Subdiffusive continuous time random walk

CTRWs were introduced by Montroll, Weiss, Scher, and Shlesinger and are jump processes characterised by random waiting times *τ* in between successive jumps^7, 8, 69–71^. These *τ* are assumed to be distributed according to the probability density function *ψ*(*τ*). As long as the mean waiting time $\langle \tau \rangle ={\int }_{0}^{\infty }\tau \psi (\tau )d\tau$ remains finite, the associated CTRW process renormalises to a discrete time random walk with absolutely sharp distribution *ψ*(*τ*) = *δ*(*τ* − 〈*τ*〉) of waiting times for sufficiently long times $t\gg \langle \tau \rangle$ ^72^. However, once a power-law form $\psi (\tau )\simeq {\tau }_{0}{(\tau /{\tau }_{0})}^{-1-\alpha }$ with 0 < *α* < 1 is chosen the characteristic waiting time 〈*τ*〉 diverges and *ψ*(*τ*) is scale free. The resulting process describes anomalous diffusion of the form (1). Note that this is consistent with the form $\psi (u)\simeq 1-{(u{\tau }_{0})}^{\alpha }$ in Laplace space^73^ which for 0 < *α* < 1 relates to the characteristic function of a completely one-sided Lévy stable law, and for *α* = 1 corresponds to *ψ*(*τ*) = *δ*(*τ* − *τ*_0_) with *τ*_0_ = 〈*τ*〉^72, 73^. A power-law form for the waiting time density in terms of *τ* is sometimes chosen, and in that case the characteristic waiting diverges marginally for *α* = 1. However, in our formulation based on the Shlesinger-Hughes idea in Laplace space, the limit *α* = 1 with the finite mean waiting time results consistently.

Power-law waiting time densities are intimately connected with random energy landscapes with exponential distribution of depths^7^, comb models^74^, weakly chaotic and turbulent systems^75–77^, as well as dynamic maps^78–80^. In experiments they are widely observed^4^. Subdiffusive CTRWs are non-stationary and weakly non-ergodic in the sense that the inequivalence $\overline{{\delta }^{2}({\rm{\Delta }})}\ne \langle {x}^{2}({\rm{\Delta }})\rangle$ remains true at arbitrarily long times^4, 37, 38^.

In the limit of many jumps subdiffusive CTRWs are characterised by the amplitude scatter distribution^38^

19 $$\mathop{{\rm{l}}{\rm{i}}{\rm{m}}}\limits_{t\to {\rm{\infty }}}\varphi (\xi )=\frac{{{\rm{\Gamma }}}^{1/\alpha }(1+\alpha )}{\alpha {\xi }^{1+1/\alpha }}{l}_{\alpha }(\frac{{{\rm{\Gamma }}}^{1/\alpha }(1+\alpha )}{{\xi }^{1/\alpha }}),$$

where *l*_*α*_(*z*) is a one-sided Lévy stable law with the Laplace image *exp*(−*u* ^*α*^)^4^.

**Figure 7:**
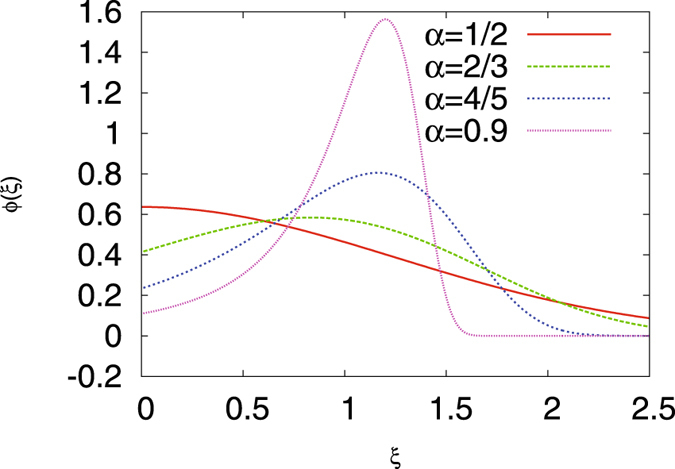
Distribution *ϕ*(*ξ*) of the amplitude scatter variable *ξ* of CTRW according to Eq. (50) or Eq. (51), respectively for the indicated *α*, calculated with Mathematica.

Figure 7 shows the amplitude scatter distribution *ϕ*(*ξ*) for various *α*. In particular, the high degree of asymmetry of the shape of *ϕ*(*ξ*) becomes obvious: even for quite large *α* the maximum of *ϕ*(*ξ*) stays to the right of the ergodic value *ξ* = 1. In the Brownian limit the distribution (19) converges to a *δ*-peak, *ϕ*(*ξ*) = *δ*(*ξ* − 1)^38, 47^, reflecting the fact that all realisations have the same, fully reproducible amplitude.

Due to the relation between the Mittag-Leffler distribution *ρ*_*α*_(*x*) and the one-sided Lévy stable law

20 $${\rho }_{\alpha }(x)=\frac{1}{\alpha }{x}^{-(1+1/\alpha )}{l}_{\alpha }({x}^{-1/\alpha }),$$

where the Mittag-Leffler function *E*_*α*_ is defined by

21 $${\int }_{0}^{\infty }{e}^{-sx}{\rho }_{\alpha }(x)dx={E}_{\alpha }(-s)=\sum _{n=0}^{\infty }\frac{{(-s)}^{n}}{{\rm{\Gamma }}\mathrm{(1}+\alpha n)}$$

with *s* ≥ 0 and 0 < *α* < 1, we can rewrite relation (19) as

22 $$\mathop{{\rm{l}}{\rm{i}}{\rm{m}}}\limits_{t\to {\rm{\infty }}}\varphi (\xi )=\frac{1}{{\rm{\Gamma }}(1+\alpha )}{\rho }_{\alpha }(\frac{\xi }{{\rm{\Gamma }}(1+\alpha )}).$$

Using the moment-generating function *M*_*ξ*_(*s*) = *E*_*α*_(−*s*Γ(1 + *α*)) the moments can be generated by

23 $$\langle {\xi }^{k}\rangle =\frac{k!{{\rm{\Gamma }}}^{k}\mathrm{(1}+\alpha )}{{\rm{\Gamma }}\mathrm{(1}+\alpha k)}.$$

Explicitly, we thus obtain the four lowest order moments in the form

24 $$\begin{array}{rcl}\langle \xi \rangle  & = & 1\\ \langle {\xi }^{2}\rangle  & = & 2{\Gamma }^{2}\mathrm{(1}+\alpha )/{\rm{\Gamma }}\mathrm{(1}+2\alpha ),\\ \langle {\xi }^{3}\rangle  & = & 6{\Gamma }^{3}\mathrm{(1}+\alpha )/{\rm{\Gamma }}\mathrm{(1}+3\alpha ),\\ \langle {\xi }^{4}\rangle  & = & 24{\Gamma }^{4}\mathrm{(1}+\alpha )/{\rm{\Gamma }}\mathrm{(1}+4\alpha \mathrm{).}\end{array}$$

Consequently the EB parameter is given by

25 $${\rm{EB}}=\frac{2{\Gamma }^{2}\mathrm{(1}+\alpha )}{\Gamma \mathrm{(1}+2\alpha )}-1,$$

consistent with the result in ref. 38, and the skewness parameter assumes the form

26 $$\gamma =\frac{6\frac{{{\rm{\Gamma }}}^{3}(1+\alpha )}{{\rm{\Gamma }}(1+3\alpha )}-6\frac{{{\rm{\Gamma }}}^{2}(1+\alpha )}{{\rm{\Gamma }}(1+2\alpha )}+2}{{(2\frac{{{\rm{\Gamma }}}^{2}(1+\alpha )}{{\rm{\Gamma }}(1+2\alpha )}-1)}^{3/2}}.$$

For the kurtosis we obtain

27 $${\mathcal{K}}=\frac{24\frac{{{\rm{\Gamma }}}^{4}(1+\alpha )}{{\rm{\Gamma }}(1+4\alpha )}-24\frac{{{\rm{\Gamma }}}^{3}(1+\alpha )}{{\rm{\Gamma }}(1+3\alpha )}+12\frac{{{\rm{\Gamma }}}^{2}(1+\alpha )}{{\rm{\Gamma }}(1+2\alpha )}-3}{{(2\frac{{{\rm{\Gamma }}}^{2}(1+\alpha )}{{\rm{\Gamma }}(1+2\alpha )}-1)}^{2}}.$$

**Figure 8:**
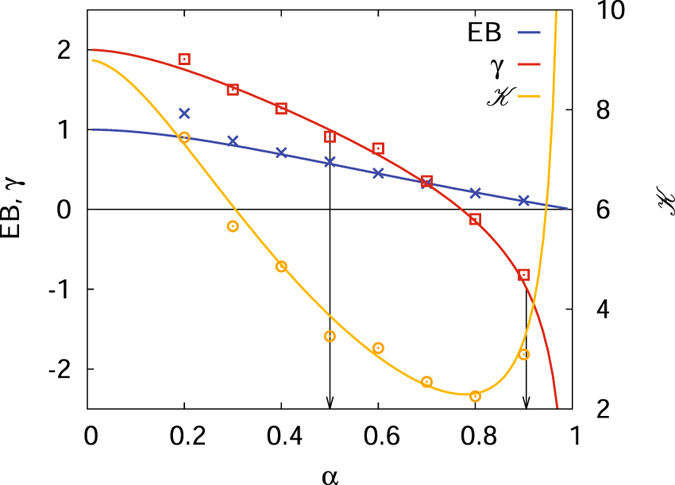
EB parameter (25), skewness (26), and kurtosis (27) (right ordinate) of the subdiffusive CTRW as a function of the anomalous diffusion exponent *α*. The solid lines represent the analytical results and the dots are the numerical results from a simulated CTRW based on ref. 115 with parameters Δ*s* = 10^−4^, Δ*t* = 10^−3^, *N*_Δ*t*_ = 10^7^, ensemble averages are taken over 10^3^ trajectories. The arrows mark the *α* values for which the absolute skewness crosses unity and are thus considered significant. Results from a discrete time random walk with Gaussian jump length distribution can be found in Fig. 9. Convergence to the limiting values *γ* = 0 and ${\mathcal{K}}=3$ for Brownian motion^47^ can be clearly anticipated.

**Figure 9:**
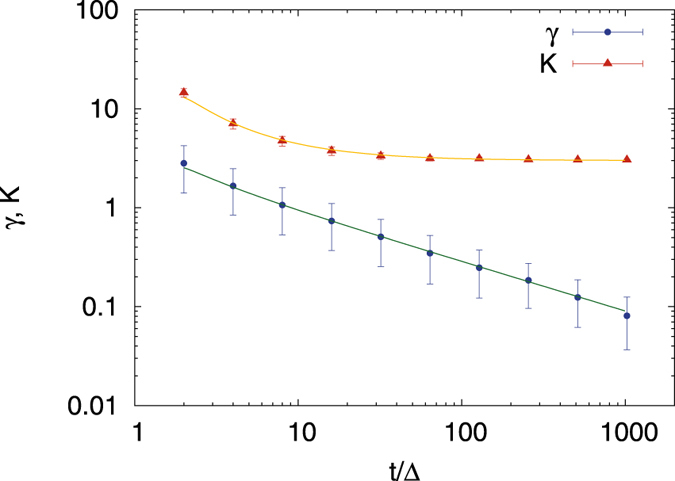
Skewness and kurtosis for a discrete-time random walk with Gaussian jump length distribution. Solid lines represent the analytic result for Brownian motion from^47^. The convergence to *γ* = 0 and ${\mathcal{K}}=3$ is nicely displayed by the data. While the skewness exhibits a power-law decay to zero the kurtosis appears to converge to its value much earlier and then does not change much any more.

Figure 8 shows the EB parameter, the skewness, and the kurtosis of the subdiffusive CTRW as a function of the anomalous diffusion exponent *α*. The EB parameter shows a relatively moderate variation over the interval *α*∈(0,1] and decays monotonically from EB = 1 at *α* → 0 to EB = 0 in the Brownian case *α* = 1. In the Brownian limit *α* = 1 corresponding to Brownian motion with finite moments 〈*τ* ^*k*^〉 of the waiting times the skewness and kurtosis reduce to *γ* = 0 and ${\mathcal{K}}=3$, respectively. The approach to these Gaussian values for finite *t*/Δ is detailed in Fig. 9. Note once more that according to our choice for the waiting time density starting from the expression $\psi (u)\sim 1-{(u{\tau }_{0})}^{\alpha }$ in Laplace space the form *ψ*(*τ*) = *δ*(*τ* − *τ*_0_) emerges in the Brownian limit, for which all moments are indeed finite.

Let us now turn to the subdiffusive case with 0 < *α* < 1. The skewness *γ* in Fig. 8 decays monotonically from *γ* = 2 in the limit *α* → 0 and generally has a more complex form than EB. *γ* changes its sign at around *α* = 0.78 meaning that the scatter distribution switches from a right-skewed to a left-skewed distribution. For most of the range of the anomalous diffusion exponent the absolute value of the skewness is greater than unity–the two arrows in Fig. 8 indicate where *γ* crosses unity–and thus significantly skewed. In this region the EB parameter is thus not a sufficient quantity to characterise the shape of the amplitude scatter distribution *ϕ*(*ξ*). When we gradually approach the Brownian case *α* = 1 we see that *γ* diverges to negative values and apparently does not converge to its zero Brownian value. The reason for this lies in the very shape of *ϕ*(*ξ*) shown in Fig. 7. Namely, the maximum of *ϕ*(*ξ*) is not centred at the ergodic value *ξ* = 1. This off-centre behaviour persists until we fully reach the Brownian case *α* = 1. When *ϕ*(*ξ*) is sufficiently sharp such that we can evaluate the moments as if they were generated by a *δ*-function positioned at *ξ* = 1 + *ε*, we obtain 〈*ξ* ^*k*^〉 = (1 + *ε*)^*k*^. In the limit *ε* → 0 the skewness (5) indeed diverges to minus infinity. This heuristic argument is consistent with the limiting behaviour of expression (26) when we approach the Brownian case: ${\mathrm{lim}}_{\alpha \to 1}\gamma =(\alpha -\mathrm{1)}/{\mathrm{(1}-\alpha )}^{\mathrm{3/2}}$. In contrast to the EB parameter, the skewness thus exhibits a discontinuous behaviour when we approach the Brownian limit. This is an important observation with respect to the analysis of subdiffusive CTRW processes. Note that in experiments values of *α* ≈ 0.9 are indeed observed and significant^26^.

Similarly, the kurtosis also varies from an intermediate value for small *α* over a minimum, reaching quite large values when the anomalous diffusion exponent approaches unity. This divergent behaviour and the discontinuity at the point *α* = 1 can be rationalised analogously to the discussion of the skewness.

In comparison to FBM we note that for the subdiffusive CTRW we consider the shape parameters at *t* → ∞. Interestingly for both the EB parameter and the skewness the general trend is opposite: for FBM the smallest values occur for low *α*, while for the CTRW this corresponds to the largest values (not taken the divergencies for larger *α* into account). The higher order moments may therefore be good inference indicators for the CTRW mechanism. We also note that due to the larger variation of the skewness with *α* it may provide a useful tool to extract the anomalous diffusion exponent from sufficiently good data. The delicate variation of the kurtosis around *α* ≤ 1 makes it a particularly good indicator for *α* in this region.

The exact variation of *γ* and ${\mathcal{K}}$ with *α* and, in particular, their apparent divergence for *α* → 1 and discontinuous behaviour at *α* = 1 are our third main result.

### Ageing CTRW

Often, the start of a measurement does not coincide with the initiation of the time evolution of the system. For instance, when we measure the motion of an endogenous lipid granule in a living cell, it is not clear what the starting point for this process is. Or, we could probe the charge current in amorphous semiconductors and on purpose introduce a time shift between the creation of the charge carriers and the moment when we switch on the driving electrical field^81, 82^. If the dynamics is stationary, a delay between system initiation and start of the measurement does not make any difference, as the correlation functions solely depend on the time difference. In a non-stationary system, in contrast, we observe ageing phenomena^4^, similar to those in glassy systems^83, 84^. The delay between initiation and start of the measurement is called the ageing time *t*_*a*_^4^.

Subdiffusive CTRWs represent a prototype case to study ageing effects^85–89^. It was shown that ageing in subdiffusive CTRWs leads to a population splitting into a fraction of particles, that never move during an observation period of duration *t* starting after the system has been allowed to age for the period *t*_*a*_ – and a fraction of variable mobility^88, 89^. For the aged process the time averaged MSD is calculated via^88, 89^

28 $$\overline{{\delta }_{a}^{2}({\rm{\Delta }})}=\frac{1}{t-{\rm{\Delta }}}{\int }_{{t}_{a}}^{{t}_{a}+t-{\rm{\Delta }}}{[x(t+{\rm{\Delta }})-x(t)]}^{2}{\rm{d}}t.$$

The distribution *ϕ*(*ξ*) for the ageing CTRW then yields in the form^88, 89^

29 $$\begin{array}{ccc}\varphi (\xi ) & \sim  & [1-{m}_{\alpha }(t/{t}_{a})]\delta (\xi )+{m}_{\alpha }(t/{t}_{a}){\rm{\Gamma }}(2-\alpha )\\  &  & \times \frac{{(t/{t}_{a})}^{1-\alpha }}{{\rm{\Gamma }}(\alpha )}{H}_{1,1}^{1,0}[\xi \frac{{(t/{t}_{a})}^{1-\alpha }}{{\rm{\Gamma }}(\alpha )}|\begin{array}{c}(2-2\alpha ,\alpha )\\ (0,1)\end{array}],\end{array}$$

where *m*_*α*_ is the probability to observe at least one jump during the observation period *t* ^88, 89^,

30 $${m}_{\alpha }(t/{t}_{a})=\frac{B({[1+{t}_{a}/t]}^{-1};1-\alpha ,\alpha )}{[\Gamma \mathrm{(1}-\alpha )\Gamma (\alpha )]},$$

in terms of the incomplete Beta function^90^ with the power-law form ${m}_{\alpha }(t/{t}_{a})\simeq {(t/{t}_{a})}^{1-\alpha }$ ^88, 89^. The resulting EB parameter is, according to refs 88, 89

31 $${\rm{EB}}=2\alpha \frac{B({[1+{t}_{a}/t]}^{-1}\mathrm{;1}+\alpha ,\alpha )}{{[1-{(1+t/{t}_{a})}^{-\alpha }]}^{2}}-1.$$

In analogy to the procedure outlined in Methods we determine the moments of *ξ* via the Mellin transform of the scatter distribution (29), yielding

32 $$\langle {\xi }^{k}\rangle ={m}_{\alpha }(t/{t}_{a}){(\frac{{\rm{\Gamma }}(\alpha )}{{(t/{t}_{a})}^{1-\alpha }})}^{k}\frac{{\rm{\Gamma }}(k+1){\rm{\Gamma }}(2-\alpha )}{{\rm{\Gamma }}(2+\alpha (k-1))}.$$

**Figure 10:**
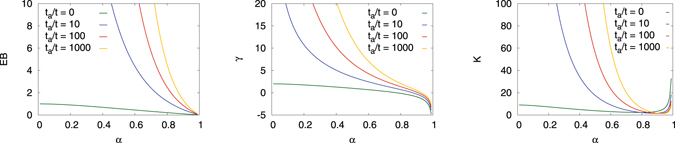
EB parameter, skewness, and kurtosis of ageing CTRW as a function of the anomalous diffusion exponent *α* in case of different ratios *t*_*a*_/*t* of the ageing time-to-measurement time. For comparison, we also include the behaviours of the parameters in the non-aged regime.

Figure 10 shows the EB parameter, skewness, and kurtosis for CTRW for different ageing time-to-measurement time ratios. When the ageing time becomes more dominant we observe an increase of the degree of non-ergodicity, due to the increasing inhomogeneity of the jump statistic following the above-mentioned population splitting^88, 89^. Except for a small parameter range of larger *α* the distribution is significantly asymmetric, as shown by the skewness parameter. Also outliers are more pronounced for an aged system, as can be seen for the kurtosis. These results for the shape parameters of the ageing process complete our study of CTRWs.

### Heterogeneous diffusion process and gamma-distributed amplitude scatter distributions

Another important class of anomalous stochastic processes are heterogeneous diffusion processes (HDPs), which may be defined in terms of the multiplicative Langevin equation^91^

33 $$\frac{{\rm{d}}x}{{\rm{d}}t}=\sqrt{2D(x)}\times \zeta (t),$$

in which *ζ*(*t*) represents Gaussian, delta-correlated noise with zero mean. We here briefly discuss the properties of HDPs for completeness. One of the well studied cases for the (quenched) position dependent diffusion coefficient is the power-law form^91^

34 $$D(x)={D}_{0}|x+{x}_{{\rm{off}}}{|}^{\beta },$$

where *D*_0_ has the dimension cm^2−*β*^ sec^−1^. The small shift *x*_off_ = 10^−2^ prevents the particle from being trapped at *x* = 0. In the Stratonovich interpretation the process was studied in detail in refs 91–93. It is known that the Stratonovich interpretation of the Langevin equation produces a so-called spurious drift, whereas a thermodynamically consistent interpretation follows from the ‘isothermal’ discretisation^94–96^. Note that rigorous results on the general symmetries of overdamped multiplicative white noise processes, clarifying some prevailing misconceptions on the Itô-Stratonovich dilemma and presenting an exact mapping of the different interpretations to a purely additive white noise process, were reported only recently^97^. Nevertheless, the notion of a ‘correct’ interpretation of multiplicative white noise Langevin equations is always context dependent^98–100^, as it was shown, for example, that general non-linear relaxation processes not satisfying a fluctuation-dissipation theorem in fact obey a Fokker-Planck equation following from the Itô interpretation of the underlying Langevin equation^101^. Moreover, as we here advocate the general importance of higher-order statistics in the quantification of stationary and/or ergodic properties of single-particle time series data, the specific interpretation of the multiplicative white noise Langevin equation is not critical. A particle in the diffusivity field *D*(*x*) is effectively pushed into regions of ever smaller diffusivity. The MSD of the process follows the power-law form (1) with *α* = 2/(2 − *β*)^91^.

**Figure 11:**
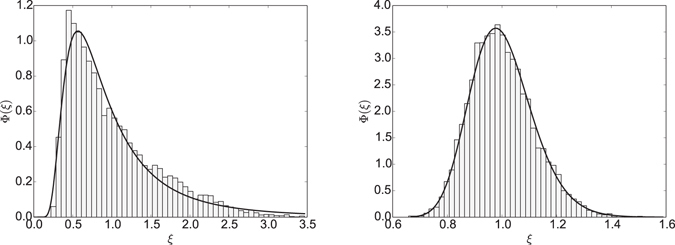
Scatter distribution of a heterogeneous diffusion process with parameters *D*_0_ = 1, trajectory length *t* = 10^4^, number of trajectories *N* = 10^4^. Left figure: *β* = 1, parameters of the fit: *a* = 2.31, *b* = 1.28 × 10^6^, *ν* = −3.08. Right figure: *β* = −1, parameters of the fit: *a* = 76.91, *b* = 1.85 × 10^3^, *ν* = −77.72.

Figure 11 shows two representative probability density functions of the HDP amplitude scatter generated by the simulation scheme

35 $${x}_{i+1}-{x}_{i}=\sqrt{2D(\frac{{x}_{i+1}+{x}_{i}}{2})}\times ({\xi }_{i+1}-{\xi }_{i}).$$

This probability density function can be fitted by the generalised Gamma distribution^91^

36 $$\varphi (\xi )=\frac{{\xi }^{\nu -1}}{2(ab{)}^{\nu /2}{K}_{\nu }(2\sqrt{a/b})}\times \exp (-\frac{a}{\xi }-\frac{\xi }{b})$$

with three fitting parameters *a*, *b* and *ν* and *ξ* > 0. Here *K*_*ν*_ is the modified Bessel function of the second kind^90^. This distribution has exponential cutoffs for both small and large argument, as specified by the parameters *a* and *b*. In between, it exhibits power-law scaling with exponent *ν* − 1. This form is similar to generic first passage densities of stochastic processes^102^. The above density has the moments^103^

37 $$\langle {\xi }^{k}\rangle ={(ab)}^{k/2}\frac{{K}_{\nu +k}(2\sqrt{a/b})}{{K}_{\nu }(2\sqrt{a/b})},$$

From which the EB parameter, the skewness, and the kurtosis can be readily constructed. Investigating the respective quantities numerically shows that the skewness can reach values larger than unity and the kurtosis may attain very large values, pointing at significant outliers in *ϕ*(*ξ*).

## Discussion

We presented an analysis of the amplitude scatter distribution of the time averaged MSD of various, widely used anomalous diffusion processes: FBM is an ergodic process in the sense that time and ensemble averages of physical observables converge to each other. Subdiffusive CTRW and HDPs are weakly non-ergodic and show a disparity between time and ensemble averages^4^. In all cases we identified relevant situations when the amplitude scatter distribution is not sufficiently characterised by the ergodicity breaking parameter EB due to the significant skewness of the distribution. A rough criterion for when the skewness is non-negligible is given when the absolute value of the skewness exceeds unity. As important additional parameters for the quantitative description of the amplitude scatter of the time averaged MSD, we analysed this skewness as well as the kurtosis of these processes. We believe that the results presented here are important additional characteristics for the analysis of anomalous stochastic time series measured in experiments and simulations in complex systems. Moreover they demonstrate the importance of the higher order moments of *ϕ*(*ξ*) in the analysis of data garnered from experiments or simulations. The amplitude scatter distribution *ϕ*(*ξ*) is a very useful quantity to classify anomalous stochastic processes. Higher order moments, as shown here, provide even more relevant information for the physical classification and interpretation of stochastic data. We note that, indeed, following better data from experiments, higher order moments provide useful information for various processes, for instance, shown in a recent study of enzyme kinetics^104^.

Specifically for FBM we revisited the EB parameter and showed that with a less severe approximation EB is continuous over the entire range of the anomalous diffusion exponent, 0 < *α* < 2, and no singularity at *α* = 3/2 occurs. We showed that the previous results are in good agreement with the ones found here sufficiently far away from *α* = 3/2. We also showed that while a previous assumption that the amplitude scatter distribution of FBM is Gaussian for short lag times is approximately correct for subdiffusive FBM, for anomalous diffusion exponents in the superdiffusive range strong asymmetries of the scatter distribution arise for which the Gaussian description becomes inadequate and the skewness therefore is an important parameter to quantify the distribution. Cognisance of this fact is particularly important for generalisations of first passage processes in chemical and molecular biological contexts^105–107^, especially when active modes are present^108, 109^. Note that indeed in ref. 34 the superdiffusive transport inside amoeba cells was shown to be of FBM form.

As parameter inference from stochastic time series is being increasingly recognised as an important field in the theory of stochastic processes, analyses demonstrate that it is vital to compare several complementary measures for a given time series, in order to identify the very physical nature of the underlying stochastic mechanism (FBM, HDP, CTRW, etc.) and correctly infer the parameters in this process^4^. Apart from the ensemble and time averaged MSDs, the EB parameter as well as the amplitude scatter distribution *ϕ*(*ξ*) and its shape parameters *γ* and ${\mathcal{K}}$ analysed in this work we mention ratios of ensemble moments and the mean maximal excursion method^110^, fractional order moments^33^, the p-variation method^54–56^, Bayesian ranking^111^, or ageing analysis^13, 14, 26–29^, see also the review^112^ on analysis of diffusive motion. Generally, using several complementary methods improves the quality of the data inference. As we show here *γ* and ${\mathcal{K}}$ are relevant parameters to distinguish stochastic processes from each other but also to extract good estimates for the anomalous diffusion exponent *α* itself, due to their high *α*-sensitivity.

An alternative approach is to use the time averaged van Hove cross-correlation functional^22^

38 $${G}_{tt}(\chi ,{\rm{\Delta }})=\frac{1}{T-{\rm{\Delta }}}{\int }_{0}^{T-{\rm{\Delta }}}\frac{\delta (|{\rm{r}}(t+{\rm{\Delta }})-{\rm{r}}(t)|-\chi )}{4\pi {\chi }^{2}}{\rm{d}}t.$$

Here 4*πχ* ^2^ *G*_*tt*_(*χ*,Δ) is the probability density to find the particle at time Δ + *t*′ at a relative displacement between *χ* and *χ* + d*χ*. This cross-correlation function essentially describes the frequency of occurrences of jump lengths *χ* along the trajectory during the lag time delta. Analysing this distribution for various values of Δ provides additional information about the process. Especially the physical aspects underlying the bimodality of the scatter distribution for aged CTRW processes might be visible in an analysis of the van Hove cross-correlation function, an aspect requiring substantial simulations work in the future.

## Methods

### Calculation of the EB parameter for FBM

We start with Eq. (11). Introducing *τ* = *t*′/Δ the integrand simplifies to terms of (1 + *τ*)^*α*^ and |1 − *τ*|^*α*^,

39 $$\begin{array}{ccc}{\rm{E}}{\rm{B}} & = & \frac{1}{t/{\rm{\Delta }}-1}{\int }_{0}^{t/{\rm{\Delta }}-1}{[{(1+\tau )}^{\alpha }+|1-\tau {|}^{\alpha }-2{\tau }^{\alpha }]}^{2}{\rm{d}}\tau \\  &  & -\,\frac{1}{{(t/{\rm{\Delta }}-1)}^{2}}{\int }_{0}^{t/{\rm{\Delta }}-1}{[{(1+\tau )}^{\alpha }+|1-\tau {|}^{\alpha }-2{\tau }^{\alpha }]}^{2}\tau {\rm{d}}\tau .\end{array}$$

We expand the integrands using the generalised binomial series

40 $${(1+x)}^{\alpha }=\sum _{n=0}^{{\rm{\infty }}}(\begin{array}{c}\alpha \\ n\end{array}){x}^{n},$$

which converges absolutely for |*x*| < 1. In order to assure an absolute convergence for all Δ/*t* as well as all *α*, we rewrite the integral as

41 $${\int }_{0}^{t/{\rm{\Delta }}-1}\ldots {\rm{d}}{\tau }^{{\rm{^{\prime} }}}=\mathop{{\rm{l}}{\rm{i}}{\rm{m}}}\limits_{\varepsilon \to 0}[{\int }_{0}^{1-\varepsilon }\ldots {\rm{d}}\tau +{\int }_{1+\varepsilon }^{\tau /{\rm{\Delta }}-1}\ldots {\rm{d}}\tau ].$$

Explicitly, Eq. (11) becomes

42 $$\begin{array}{ccc}{\rm{E}}{\rm{B}} & = & \frac{4}{t/{\rm{\Delta }}-1}\mathop{{\rm{l}}{\rm{i}}{\rm{m}}}\limits_{\varepsilon \to 0}({\int }_{0}^{1-\varepsilon }{[\displaystyle {\sum }_{n=1}^{{\rm{\infty }}}(\begin{array}{c}\alpha \\ 2n\end{array}){\tau }^{2n}+1-{\tau }^{\alpha }]}^{2}{\rm{d}}\tau \\  &  & +{\int }_{1+\varepsilon }^{t/{\rm{\Delta }}-1}[\displaystyle {\sum }_{n=1}^{{\rm{\infty }}}\displaystyle {\sum }_{{n}^{{\rm{^{\prime} }}}=1}^{{\rm{\infty }}}(\begin{array}{c}\alpha \\ 2n\end{array})(\begin{array}{c}\alpha \\ {2}^{{\rm{^{\prime} }}}\end{array}){\tau }^{-2(n+{n}^{{\rm{^{\prime} }}}-\alpha )}]{\rm{d}}\tau )\\  &  & -\frac{4}{{(\frac{t}{{\rm{\Delta }}}-1)}^{2}}\mathop{{\rm{l}}{\rm{i}}{\rm{m}}}\limits_{\varepsilon \to 0}({\int }_{0}^{1-\varepsilon }{[\displaystyle {\sum }_{n=1}^{{\rm{\infty }}}(\begin{array}{c}\alpha \\ 2n\end{array}){\tau }^{2n}+1-{\tau }^{\alpha }]}^{2}\tau {\rm{d}}\tau \\  &  & +{\int }_{1+\varepsilon }^{t/{\rm{\Delta }}-1}[\displaystyle {\sum }_{n=1}^{{\rm{\infty }}}\sum _{{n}^{{\rm{^{\prime} }}}=1}^{{\rm{\infty }}}(\begin{array}{c}\alpha \\ 2n\end{array})(\begin{array}{c}\alpha \\ {2}^{{\rm{^{\prime} }}}\end{array}){\tau }^{-2(n+{n}^{{\rm{^{\prime} }}}-\alpha )+1}]{\rm{d}}\tau ).\end{array}$$

We interchange the integral and the sums and take the limit *ε* → 0. Note that we have not made any approximation up to this point. Focusing on ${\rm{\Delta }}/t\ll 1$ we only keep terms of leading order. As we show below, the special result for *α* = 3/2 can be obtained smoothly from this result by taking the limit *α* → 3/2 from below and from above. For *α* ≠ 3/2 after performing the integrals we obtain

43 $$\begin{array}{ccc}{\rm{E}}{\rm{B}} & = & \frac{{\rm{\Delta }}}{t}4[\displaystyle {\sum }_{n=1}^{{\rm{\infty }}}\displaystyle {\sum }_{{n}^{{\rm{^{\prime} }}}=1}^{{\rm{\infty }}}(\begin{array}{c}\alpha \\ 2n\end{array})(\begin{array}{c}\alpha \\ {2}^{{\rm{^{\prime} }}}\end{array})(\frac{1}{2(n+{n}^{{\rm{^{\prime} }}})+1}-\frac{1}{-2(n+{n}^{{\rm{^{\prime} }}}-\alpha )+1})\\  &  & +\,2\displaystyle {\sum }_{n=1}^{{\rm{\infty }}}(\begin{array}{c}\alpha \\ 2n\end{array})(\frac{1}{2n+1}-\frac{1}{2(n+\frac{\alpha }{2})+1})+\frac{2\alpha +2}{2\alpha +1}-\frac{2}{\alpha +1}]\\  &  & +\,4\displaystyle {\sum }_{n=1}^{{\rm{\infty }}}\displaystyle {\sum }_{{n}^{{\rm{^{\prime} }}}=1}^{{\rm{\infty }}}(\begin{array}{c}\alpha \\ 2n\end{array})(\begin{array}{c}\alpha \\ {2}^{{\rm{^{\prime} }}}\end{array}){(\frac{{\rm{\Delta }}}{t})}^{2(n+{n}^{{\rm{^{\prime} }}}-\alpha )}(\frac{1}{-2(n+{n}^{{\rm{^{\prime} }}}-\alpha )+1}\\  &  & -\,\frac{1}{-2(n+{n}^{{\rm{^{\prime} }}}-\alpha )+2}).\end{array}$$

The last term (Δ/*t*)^2(*n*+*n*′−*α*)^ only becomes relevant if *n*,*n*′ = 1. The contributions of the sums decrease as *n*,*n*′ increase. Hence, being interested in ${\rm{\Delta }}/t\ll 1$ we may neglect all terms but those with *n*,*n*′ = 1. Thus, we find Eq. (12) in the main text.

Alternatively Eq. (11) can be calculated explicitly leading to the expression

44 $$\begin{array}{ccc}{\rm{E}}{\rm{B}} & = & \frac{4}{(\frac{t}{{\rm{\Delta }}}-1)}[\frac{{2}^{-(3+2\alpha )\pi }}{{\rm{\Gamma }}(-1-2\alpha ){\rm{\Gamma }}{(\frac{3}{2}+\alpha )}^{2}\,\sin \,(2\pi \alpha )}\\  &  & +\frac{25(3+2\alpha )\,{}_{3}F{}_{2}(\begin{array}{c}\frac{3}{2},\frac{3}{2}-\alpha ,-\alpha ;\\ \frac{5}{2},\frac{5}{2};\end{array}1)+2\alpha (4{\alpha }^{2}-9)\,{}_{3}F{}_{2}(\begin{array}{c}\frac{5}{2},1-\alpha ,\frac{5}{2}-\alpha ;\\ \frac{7}{2},\frac{7}{2};\end{array})}{150(1+\alpha )}\\  &  & +2(\frac{{}_{3}F{}_{2}(\begin{array}{c}-\frac{1}{2}-\alpha ,\frac{1}{2}-\frac{\alpha }{2},-\frac{\alpha }{2};\\ \frac{1}{2},\frac{1}{2}-\frac{\alpha }{2};\end{array}\,1)}{1+2\alpha }-\frac{{}_{3}F{}_{2}(\begin{array}{c}\frac{1}{2}-\frac{\alpha }{2},\frac{1}{2}+\frac{\alpha }{2},-\frac{\alpha }{2};\\ \frac{1}{2},\frac{3}{2}+\frac{\alpha }{2};\end{array}1)}{1+\alpha })\\  &  & +\displaystyle {\sum }_{n=0}^{{\rm{\infty }}}(3\alpha -1){(\frac{t}{{\rm{\Delta }}}-1)}^{1+2(\alpha -n)}(\begin{array}{c}\alpha \\ 2n\end{array})\frac{{}_{4}F{}_{3}(\begin{array}{c}\frac{1}{2}-\frac{\alpha }{2},-\frac{\alpha }{2},-\frac{1}{2}-n,-n;\\ \frac{1}{2},\frac{1}{2}+\frac{\alpha }{2}-n,1+\frac{\alpha }{2}-n;\end{array}1)}{(1+2\alpha -2n)(2(n-1)+\alpha )}\\  &  & +\frac{{(\frac{t}{{\rm{\Delta }}}-1)}^{1+2\alpha }}{({\alpha }^{2}-1)}((\alpha -1)\,{}_{3}{F}_{2}(\begin{array}{c}-1-\alpha ,\frac{1}{2}-\frac{\alpha }{2},-\frac{\alpha }{2};\\ \frac{1}{2},-\alpha ;\end{array}\frac{1}{{(\frac{t}{{\rm{\Delta }}}-1)}^{2}})\\  &  & +2(\alpha +1){}_{3}F{}_{2}(\begin{array}{c}\frac{1}{2}-\frac{\alpha }{2},-\frac{1}{2}+\frac{\alpha }{2},-\frac{\alpha }{2};\\ \frac{1}{2},\frac{1}{2}+\frac{\alpha }{2};\end{array}\frac{1}{{(\frac{t}{{\rm{\Delta }}}-1)}^{2}}))\\  &  & +{(\frac{t}{{\rm{\Delta }}}-1)}^{2\alpha +1}(\frac{1}{2\alpha +1}-\frac{1}{2\alpha +2})]\\  &  & -\frac{4}{{(\frac{t}{{\rm{\Delta }}}-1)}^{2}}[\frac{3\times {2}^{2(1+\alpha )}(4+{\alpha }^{2}+5{\alpha }^{3}+2{\alpha }^{4})+2(2+\alpha )(3+2\alpha )}{12(1+\alpha )(2+\alpha {)}^{2}(1+2\alpha )(3+2\alpha )}\\  &  & \times (3\alpha (3+\alpha )-(1+\alpha )(2+\alpha )(2\alpha -1)\,{}_{4}F{}_{3}(\begin{array}{c}2,2,\frac{3}{2}-\alpha ,-\alpha ;\\ 1,\frac{5}{2},3;\end{array}1))\\  &  & +\frac{{}_{3}F{}_{2}(\begin{array}{c}-1-\alpha ,\frac{1}{2}-\frac{\alpha }{2},-\frac{\alpha }{2};\\ \frac{1}{2},-\alpha ;\end{array}1)}{1+\alpha }-2\frac{{}_{3}F{}_{2}(\begin{array}{c}\frac{1}{2}-\frac{\alpha }{2},1+\frac{\alpha }{2},-\frac{\alpha }{2};\\ \frac{1}{2},\,2+\frac{\alpha }{2};\end{array}1)}{2+\alpha }].\end{array}$$

For the reason of clarity we decided to use the approximation above since it shows excellent agreement with the numerical results.

We note in passing that a direct use of Mathematica to evaluate the integrals leads to problematic results, as the necessary analytic continuations for the involved special functions are neglected.

### Continuous time random walk: calculation of the moments and skewness in the Brownian limit

We here consider an alternative derivation of the amplitude scatter distribution and its moments for subdiffusive CTRW in terms of a Fox *H*-function. Identifying the Laplace image of a one-sided Lévy stable density *l*_*α*_(*t*) with the corresponding *H*-function yields^113^

45 $$\exp (-{u}^{\alpha })=\frac{1}{\alpha }{H}_{0,1}^{1,0}[u|\begin{array}{c}-\\ (0,1/\alpha )\end{array}].$$

By help of the Laplace inversion formula in ref. 114 one then obtains the representation

46 $${l}_{\alpha }(t)=\frac{1}{\alpha t}{H}_{1,1}^{1,0}[\frac{1}{t}|\begin{array}{c}(0,1)\\ (0,1/\alpha )\end{array}].$$

Given the argument in *l*_*α*_ and the prefactor in Eq. (19), this means that

47 $$\varphi (\xi )=\frac{1}{\alpha \xi }{H}_{1,1}^{1,0}[\frac{\xi }{{\rm{\Gamma }}(1+\alpha )}|\begin{array}{c}(0,\alpha )\\ (0,1)\end{array}].$$

The moments of *ξ* can then be obtained as the Mellin transform of the *H*-function, which we know as the kernel of the defining Mellin-Barnes integral, see, e.g.ref. 8,

48 $$\langle {\xi }^{k}\rangle =\frac{1}{\alpha }{\int }_{0}^{{\rm{\infty }}}{\xi }^{k-1}{H}_{1,1}^{1,0}[\frac{\xi }{{\rm{\Gamma }}(1+\alpha )}|\begin{array}{c}(0,\alpha )\\ (0,1)\end{array}]=\frac{1}{\alpha }{{\rm{\Gamma }}}^{k}(1+\alpha )\chi (-k),$$

where the kernel of the *H*-function in relation (48) is ref. 113

49 $$\chi (s)=\frac{\Gamma (-s)}{\Gamma (-\alpha s)}\mathrm{.}$$

To obtain concrete forms of *ϕ*(*ξ*) in terms of simpler functions one can use the series expansion of the *H*-function^113^,

50 $$\varphi (\xi )=\frac{1}{\alpha \xi }\sum _{n=0}^{{\rm{\infty }}}\frac{{(-1)}^{n}}{n!{\rm{\Gamma }}(-\alpha n)}{(\frac{\xi }{{\rm{\Gamma }}(1+\alpha )})}^{n}.$$

By help of Mathematica this computable form of *ϕ*(*ξ*) then outputs, for instance,

51 $$\varphi (\xi )=\frac{2}{\pi }\exp (-\frac{{\xi }^{2}}{\pi })$$

for *α* = 1/2. Exact yet somewhat more complicated forms can be obtained for *α* = 2/3, 3/4, 9/10, and 19/20, to study the change of shape when *α* → 1.

### Brownian limit

If *ϕ*(*ξ*) = *δ*(*ξ* − 1) for the Brownian case expected from the fact that all moments of *ψ*(*τ*) are finite, what is then the skewness *γ* associated with it? Consider the limiting distribution

52 $$\varphi (\xi )=\frac{\sqrt{2}}{\sqrt{\pi {\sigma }^{2}}}\frac{1}{1+{\rm{e}}{\rm{r}}{\rm{f}}(1/\sqrt{2{\sigma }^{2}})}\exp (-\frac{{(\xi -1)}^{2}}{2{\sigma }^{2}}),$$

normalised on the interval [0,∞) and centred at *ξ* = 1. Generally, *γ* for this choice of *ϕ*(*ξ*) depends on the width *σ*, but for sufficiently small *σ* values the expected result ${\mathrm{lim}}_{\sigma \to 0}\gamma =0$ for a Gaussian distribution is reached. This heuristic argument to evaluate *γ* for the Brownian case is consistent with the analytic derivation in ref. 47 Similar considerations hold for the kurtosis.

Can one now explain the minus infinity in *γ* (and analogously for the kurtosis)? Consider the general expression for the skewness,

53 $$\gamma =\frac{\langle {\xi }^{3}\rangle -3\langle {\xi }^{2}\rangle +2}{{(\langle {\xi }^{2}\rangle -1)}^{3/2}}.$$

Now assume that for *α* → 1 the scatter distribution indeed converges towards the *δ* peak at *ξ* = 1, however, always stays centred a little to the right of *ξ* = 1, say *ξ* = 1 + *ε*. We then see that all moments become 〈*ξ* ^*k*^〉 = 1 + *ε*. Indeed, *γ* → −∞ as *ε* → 0. That in fact the peak is somewhat to the right of *ξ* = 1 can be seen in Fig. 7. This means that the convergence to the *δ* shape is faster than the shift of the central position towards unity.
